# Preparation and characterization of polycarbonate/multiwalled carbon nanotube nanocomposites

**DOI:** 10.3762/bjnano.8.203

**Published:** 2017-09-27

**Authors:** Claudio Larosa, Niranjan Patra, Marco Salerno, Lara Mikac, Remo Merijs Meri, Mile Ivanda

**Affiliations:** 1 Department of Civil, Chemical and Environmental Engineering, University of Genoa, via Opera Pia 15, 16145 Genoa, Italy; 2 Department of Mechanical Engineering, University of Wyoming, 82071 Laramie, USA; 3 Materials Characterization Facility, Istituto Italiano di Tecnologia, via Morego 30, 16163 Genoa, Italy; 4 Rudjer Bošković Institute, Center of Excellence for Advanced Materials and Sensing Devices, Bijenička 54, 10000 Zagreb, Croatia,; 5 Department of Polymer Materials, Riga Technical University, Azenes Str. 14/24, Riga LV-1048, Latvia

**Keywords:** multiwalled carbon nanotubes, nanocomposites, polycarbonate, thermal analysis, vibrational spectroscopy

## Abstract

A polymer nanocomposite was produced by ultrasonic-assisted dispersion of multiwalled carbon nanotubes (MWCNTs) in a polycarbonate matrix using *p*-xylene and dichloromethane as the solvents. The filler loading was varied from 1 to 3 wt % in order to examine the effect of MWCNTs on the structure and properties of the composites. The nanocomposites were characterized by DSC, DTA, TGA, UV–vis, FTIR and Raman spectroscopy to evaluate the changes induced by the filler in the polymer matrix. UV–vis, FTIR and Raman spectroscopy measurements confirmed the presence of the dispersed phase in the composite films, while TGA and DSC analysis of the nanocomposites revealed enhanced thermal stability and decreased crystallinity, respectively, as compared to the neat polymer. The proposed composites can find application in a number of everyday products where polycarbonate is the base polymer.

## Introduction

Polycarbonate (PC) is a polymer with remarkable mechanical and optical properties, broadly used for water bottles, monitor screens and aircraft interiors but also in business buildings and automotive light covers [1]. Some of these applications involve extended daylight exposure, which, especially due to UV radiation, induce progressive, irreversible changes affecting the lifetime, for example, optical polarization, which is associated with crystallization [2–3]. PC is an amorphous polymer with very low crystalline content, estimated at 1–2 wt %. Not only does UV exposure induce PC ageing, leading to crystallization, but also some solvents as well. They are thought to cause changes in chain conformation, which is associated with the presence of spherulite aggregates that are visible even under low magnification. Conventional thermoplastic processing is used to manufacture distinct PC products, where triazole compounds are commonly used as additives to stabilize PC and to retard the yellowing of PC upon exposure to light [4]. However, triazoles have serious thermal stability issues at the hot embossing temperature of PC. It has been suggested that the addition of MWCNTs can significantly change the mechanical properties of PC, as well as affect the crystallization behavior [5].

In former work, the effect of adding gold nanoparticles on the optical properties of PC was investigated [6]. Herein, we investigated the effect of MWCNT loading on the solvent-induced crystallization behavior of PC, eventually providing a better understanding that is useful for possible future control this phenomenon in PC-based consumer products. Proper filler dispersion is a common issue in obtaining good quality nanocomposites, especially in the case of high aspect ratio fillers such as CNTs. The efficiency in minimizing the amount of entangled bundles of MWCNTs and ensuring proper dispersion of them in the polymer matrices influences nearly all relevant properties of the composites [5,7]. Among the methods used for introduction of MWCNTs into the polymers [8], solution-based approaches ensure several advantages over direct melt mixing with respect to improved dispersion of the nanofillers within the polymer matrix due to lower viscosity of the dispersion media. Thus, we used *p*-xylene and dichloromethane solvent to mix MWCNTs and prevent their agglomeration induced by strong van der Waals forces. Previous work carried out on PC/MWCNT composites with focus on the mechanical properties showed an increase in the storage modulus obtained from indentation measurements at loadings as high as 10 wt % [9]. However, to the best of our knowledge, this is the first time that this composite system is investigated in detail for crystallinity and thermal stability. The research activity and procedures adopted during this work and reported here are in agreement with the predetermined objectives of the COST action Multi Comp CA15107 [10], aiming to improve the dispersion and stability of carbon-based suspensions and polymer composites.

## Experimental

### Materials

Commercial MWCNTs were used (FutureCarbon GmbH, Bayreuth, Germany), having a nominal diameter of 50 ± 20 nm and a length of 1–15 µm. PC pellets were used (Makrolon^®^, Bayer), having nominal density of 1.19 g cm^−3^, molecular weight of ≈*M*_w_ 54000 and polydispersity of 1.77. *p*-Xylene and dichloromethane of analytical grade (99.99% purity) were used (Sigma-Aldrich, Italy) without further purification.

### Nanocomposite film preparation

The pretreatment of PC, dispersion of MWCNTs and preparation of composite films was carried out using the following procedure. Since PC is a hygroscopic material with a tendency to absorb moisture from the environment, it was preheated in a vacuum oven at 120 °C for 4 h before processing. The moisture absorption was estimated at around 1–3% per day. After drying, the MWCNTs were dispersed in a solvent mixture of *p*-xylene and dichloromethane in 1:8 vol/vol at a concentration of 3 g/L and mixed using an ultrasonic bath (Fisher Scientific, FS60, Italy) operating at 40% amplitude for a total time of 30 min with on/off cycles (4 s and 2 s, respectively) in order to prevent heating which could occur during acoustic cavitation. The initial swelling of MWCNT agglomerates by solvent infiltration and interaction was considered as a crucial precondition to obtain a good dispersion of MWCNTs inside the polymer matrix [7]. Similarly, PC was separately dissolved in the same solvent mixture used to disperse MWCNTs at a concentration of 37 g/L. Then, a given amount of MWCNT dispersion was added to the PC solution in such a way as to obtain the predetermined MWCNT loading versus PC and mixed using a vortex mechanical agitator followed by sonication for thorough mixing of the two components without agglomeration. The obtained nanocomposite solutions (at different MWCNT loadings of 0, 1, 2 and 3 wt %) were cast into glass Petri dishes filled up to similar levels in order to maintain similar film thicknesses. The casted materials were allowed to dry at room temperature for 24 hours to obtain transparent nanocomposite films. The film thickness was estimated at 220 ± 50 µm, as determined by a screw gauge micrometer.

### Characterization

The dispersion of MWCNTs in PC, as achieved in the cast films, was characterized by UV–vis spectrometry using a Cary 6000i spectrometer by Varian, UK, in double beam configuration with empty reference sample position.

Fourier-transform infrared spectroscopy (FTIR) of PC/MWCNT composites with different loadings was carried out in the range of 600–4000 cm^−1^ on a Bruker Vertex 70 spectrometer, Bruker, Madison, USA. The samples were analyzed in attenuated total internal reflection absorbance mode, with an aperture diameter of 3 mm and a spectral resolution of 4 cm^−1^. For an optimal signal-to-noise ratio, 64 scans were averaged per sample spectrum and apodized. All the spectra were normalized thereafter.

Raman spectra were acquired with an inVia micro-Raman spectrometer by Renishaw, Gloucester, UK using a He–Ne laser excitation source emitting at a wavelength of 632.8 nm with a 20× objective. The data acquisition time was 30 s. The slit provided a spectral resolution of 1 cm^−1^. Instrument calibration was performed using silicon samples.

To investigate the stability, purity and thermal resistance of the materials, thermogravimetric analysis (TGA) was used [11–14] (TGA/DSC-1, Mettler-Toledo, Italy). Simultaneous differential thermal analysis (DTA) was used to characterize the nanocomposites in nitrogen (flow rate 50 mL/min) from RT to 800 °C at a ramp rate of 10 °C/min. The TGA/DTA instrument was calibrated with standard weight indium standards for DTA heat flow.

A differential scanning calorimetry (DSC) instrument (Pyris Diamond by Perkin Elmer, UK) was used to record the thermal profile. DSC measurements were performed with an initial mass of ≈3 mg. All the tests were carried out in a nitrogen atmosphere at a flow rate of 30 mL/min at a heating rate of 10 °C/min. The DSC instrument was calibrated by using In and Zn as a standard.

## Results and Discussion

### Spectroscopic analysis

**Figure 1:**
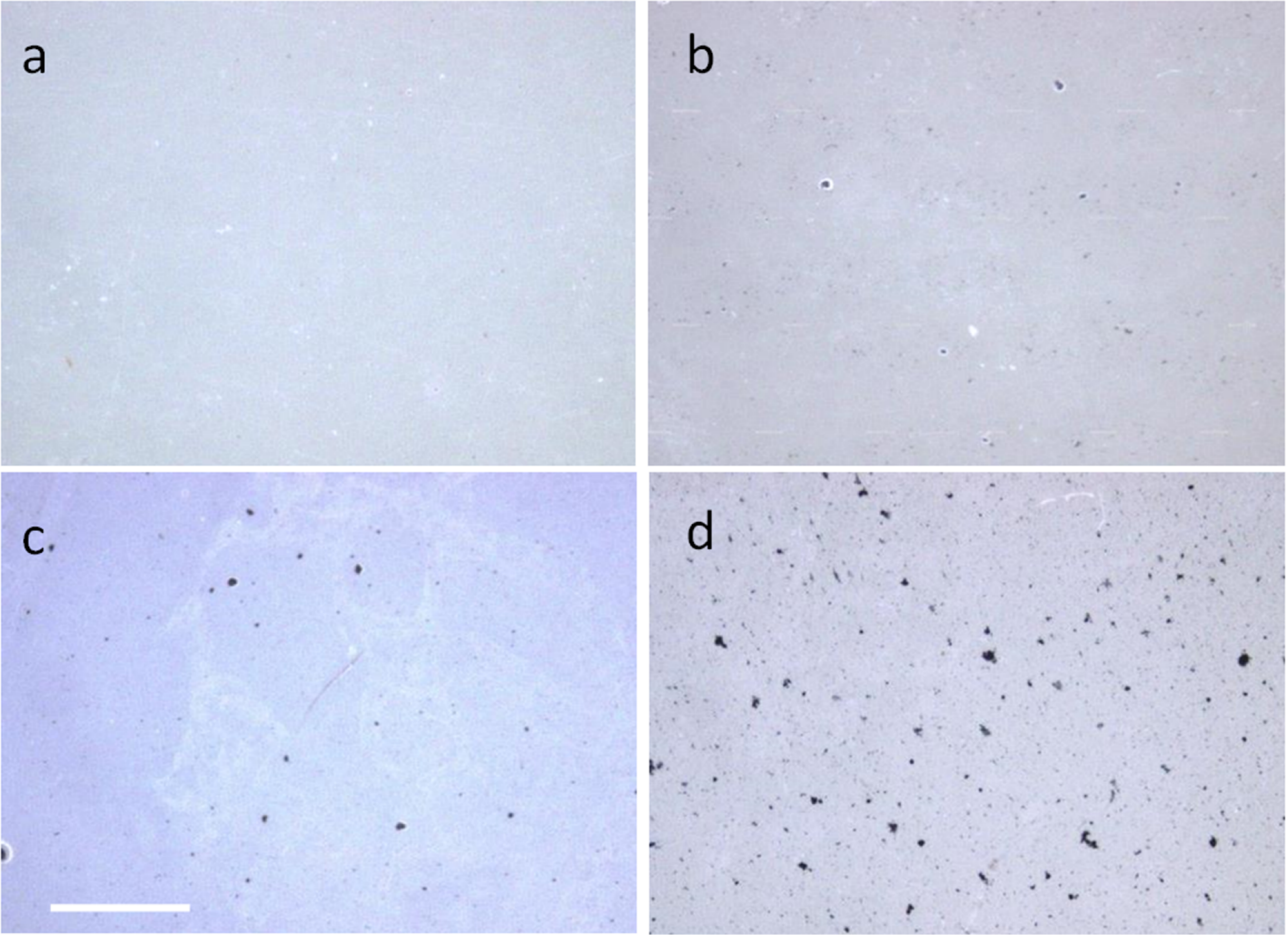
Optical stereomicroscope images (30× magnification) of PC composite films at different MWCNT loadings: a) 0, b) 1, c) 2 and d) 3 wt %. Scale bar: 100 µm, same for all images.

The typical quality of the nanocomposite films investigated in the present study is demonstrated by the low-resolution optical micrographs presented in Figure 1. The presence of occasional micrometer-scale aggregates upon drying is evident, especially at higher MWCNT loading, yet the presence of submicrometer-scale filler particles throughout the polymer matrix is also observed even at the highest MWCNT loading (see Figure 1d).

**Figure 2:**
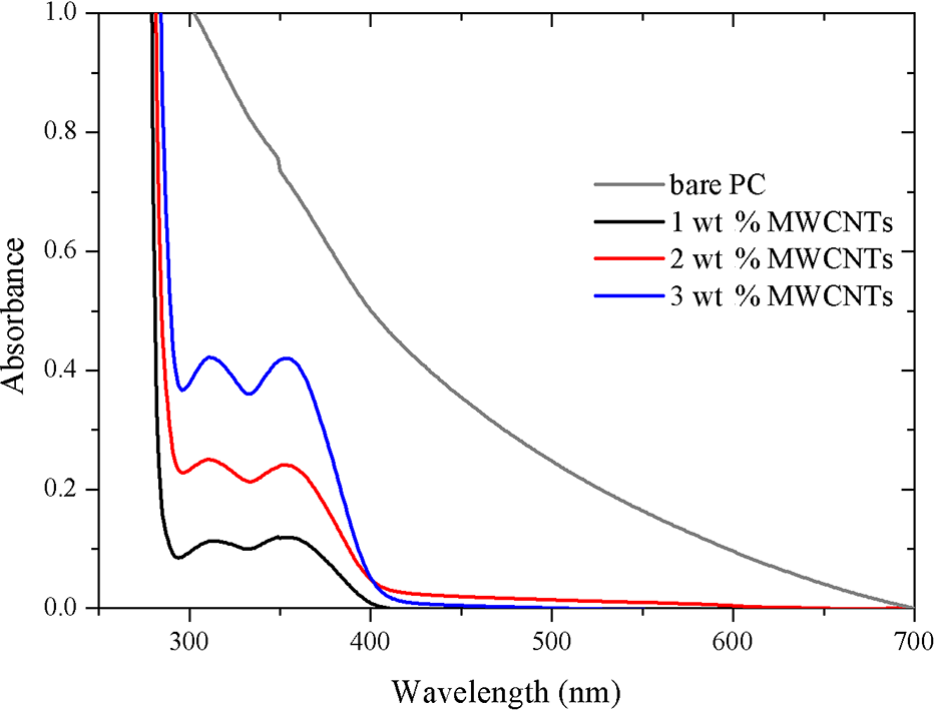
UV–vis absorbance of composite samples with different MWCNT loading.

UV–vis absorption spectra of the PC/MWCNT composite films are presented in Figure 2. Good dispersion is crucial for optimal optical properties and ensures maximum surface area for filler/polymer matrix interaction. The absorption band observed at around 289 nm could be assigned to the individual MWCNTs due to the one-dimensional van Hove singularities [15–16]. The absorbance band intensity increases with increasing filler loading with maximum absorbance observed for the composition with 3 wt % loading of MWCNTs. This absorption is characteristic of individually dispersed MWCNTs, whereas strongly bundled MWCNTs do not show an absorption band in 200–1200 nm wavelength region as their photoluminescence is quenched or the carriers are tunneling between the nanotubes [17]. Also the absorption spectrum decreases slowly in the 362–289 nm range due to scattering in the lower wavelength range. These results are an indication that the nanotubes exist as large agglomerates and are strongly entangled before sonication and that the sonication treatment in polar solvent helps to overcome the van der Waals interaction in the nanotubes, eventually leading to better dispersion.

**Figure 3:**
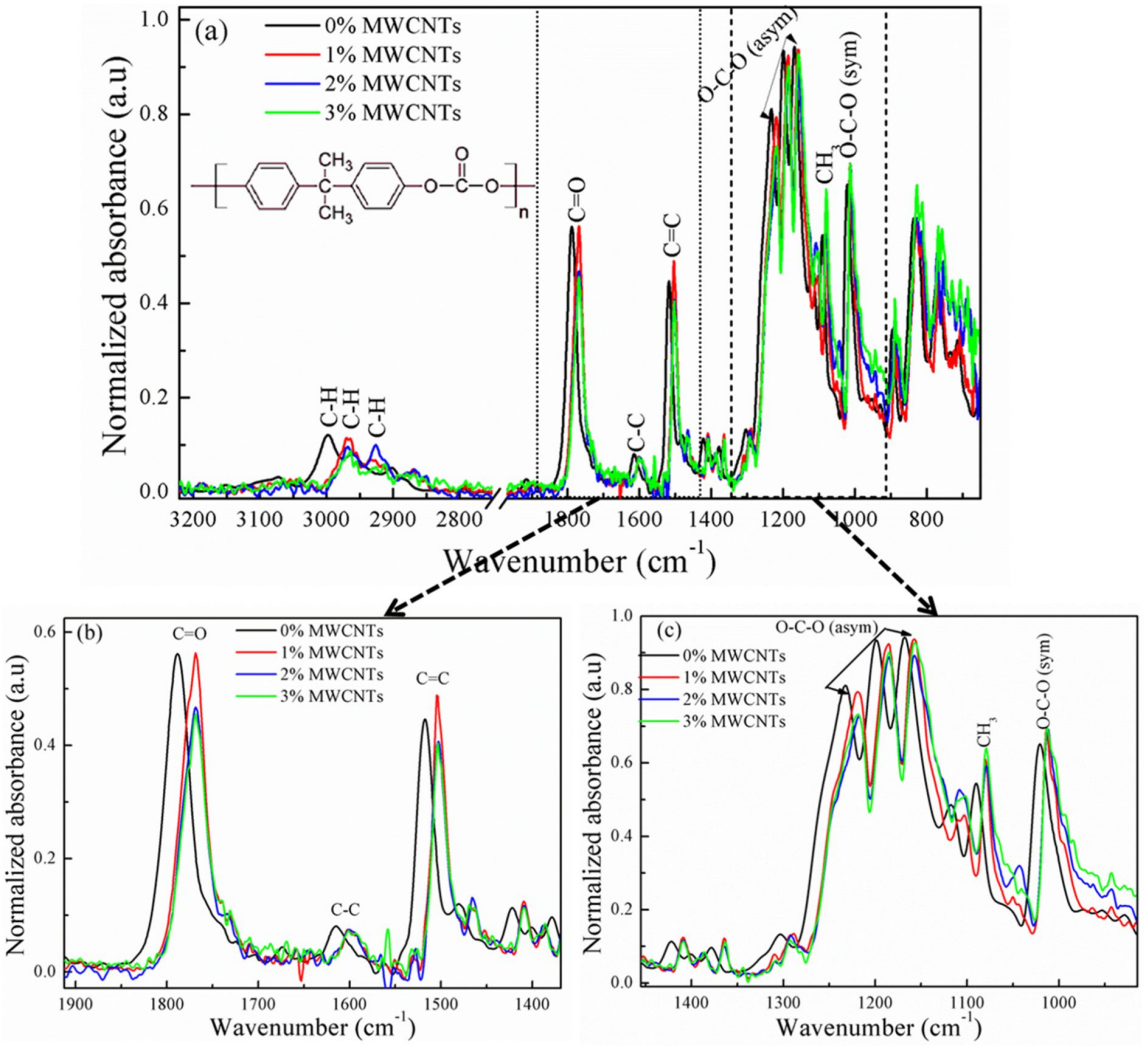
a) FTIR absorption spectrum of the PC/MWCNT composite films at different loadings (inset: structure of PC). Zoomed area in the b) carbonyl and c) ester group spectral region.

Figure 3 shows the normalized FTIR absorption spectra of principal absorbance bands of the investigated PC/MWCNT nanocomposite films. FTIR spectroscopy demonstrated to be a useful tool to investigate the structural changes in different material systems [18–20].

The characteristic IR bands of C–H from the aromatic rings are observed in the 2927 to 3000 cm^−1^ region, together with the carbonate linker/carbonyl functional group (C=O) deformation at 1790 cm^−1^. The stretching of the C–C bond from the phenyl group (benzene ring) occurs at ≈1600 cm^−1^ and the C=C bond vibration at 1504 cm^−1^. The stretching of the ester group (O–C–O) occurs from 1165 to 1232 cm^−1^ [21]. Comparing the spectral band of bare PC with the 1, 2, and 3% MWCNT-loaded nanocomposites reveals that the carbonyl band (C=O) in the spectrum of the former is shifted from 1788 to 1768 cm^−1^. The C=C band at 1518 cm^−1^ is shifted to 1504 cm^−1^ and the bands at 1233 and 1200 cm^−1^ in the region of the asymmetric O–C–O stretching vibration are transferred to a band at 1218 cm^−1^ with a shoulder at 1258 cm^−1^. The changes in the observed shift and intensity clearly indicate a change in crystallinity of the nanocomposites after nanotube loading.

**Figure 4:**
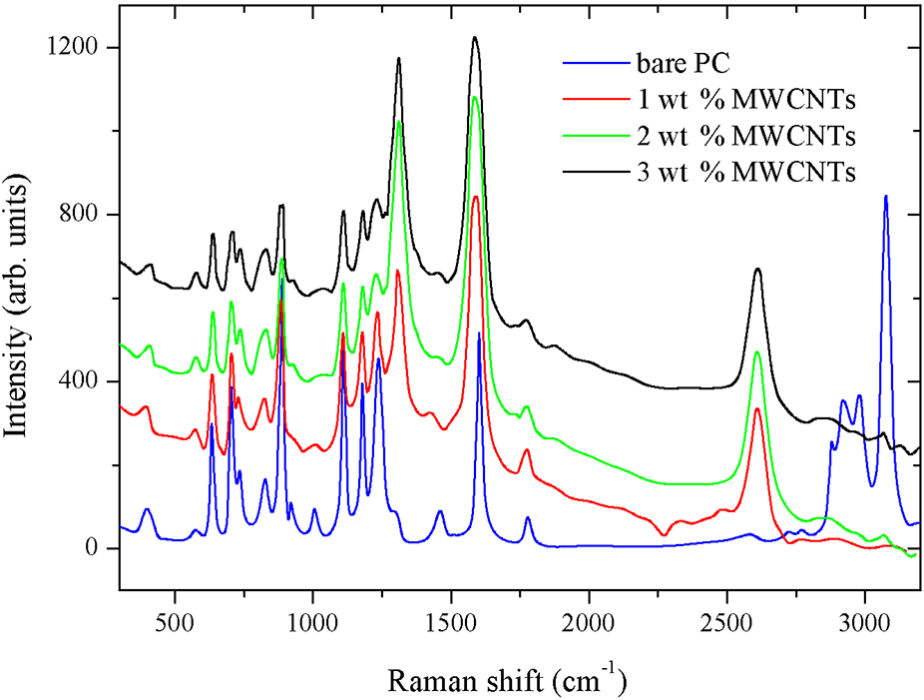
Raman spectra of PC/MWCNT composite films at different MWCNT loadings.

The Raman spectra of the different PC/MWCNT composite films are shown in Figure 4. The blue curve represents the spectrum of bare PC, with the full fingerprint of the polymer in the region around 3000 cm^−1^. The other curves show the spectra of composites with different loading, which are normalized to the G’ overtone band of MWCNTs at 2683 cm^−1^. In these spectra, the characteristic peaks for MWCNTs at 1342 cm^−1^ (D band) and 1580 cm^−1^ (G band) are clearly observed [22]. Additionally, one can see that upon increasing the MWCNT loading, the peaks characteristic of PC are depressed. Also, the peak around 1600 cm^−1^ undergoes doublet formation with overall shifting towards lower wavenumbers, which reveals the interaction between MWCNTs and PC [23].

### Thermal analysis

**Figure 5:**
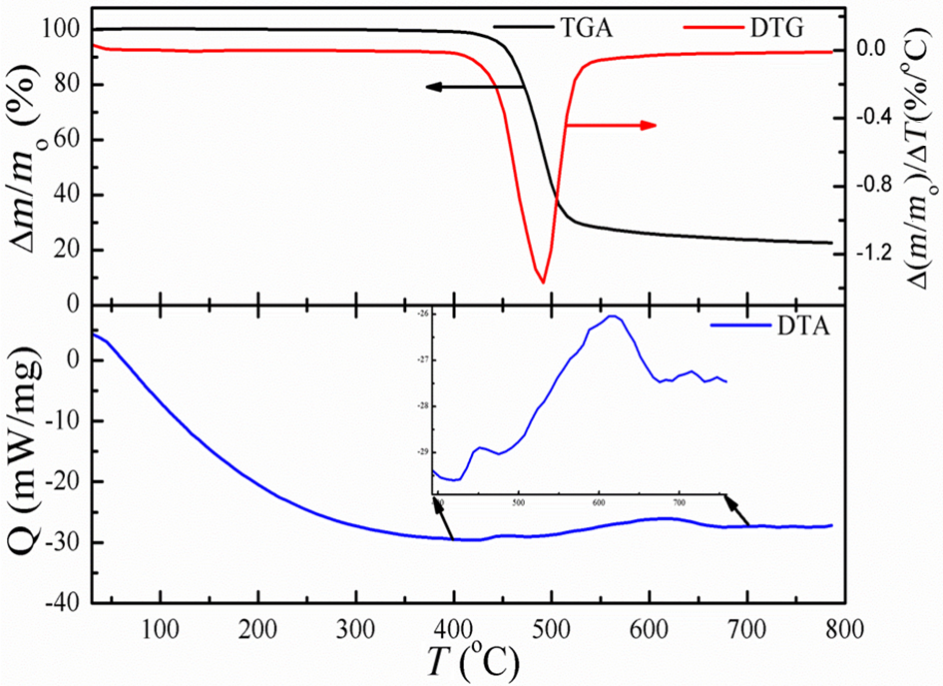
TGA/DTG and DTA analysis of the PC/MWCNT films at 1 wt % loading under N_2_ flow. The inset in the lower panel shows the zoomed image of the DTA plot in the region of highest mass loss.

Figure 5 shows the TGA/DTG and DTA analysis of the PC/MWCNT sample with 1 wt % loading. The profiles of the other composites (2 and 3 wt %) were qualitatively similar, apart from the shift in final wt % level and have not been included for the sake of clarity. No change in mass is observed in Figure 5 around 100–200 °C, indicating that the nanocomposite is free from any absorbed solvent or moisture. The major mass loss of around 80% occurred from 400 to 525 °C, and is due to the decomposition of the polymer matrix. The residual mass of 20% is comprised of both the MWCNTs and the amorphous carbon, which obviously comes from the organic matrix. DTA reveals a broad, exothermic peak followed by a small shoulder, which is believed to be due to the interaction and thermal expansion of MWCNTs in the PC matrix. No other thermal changes are observed after 700 °C.

**Figure 6:**
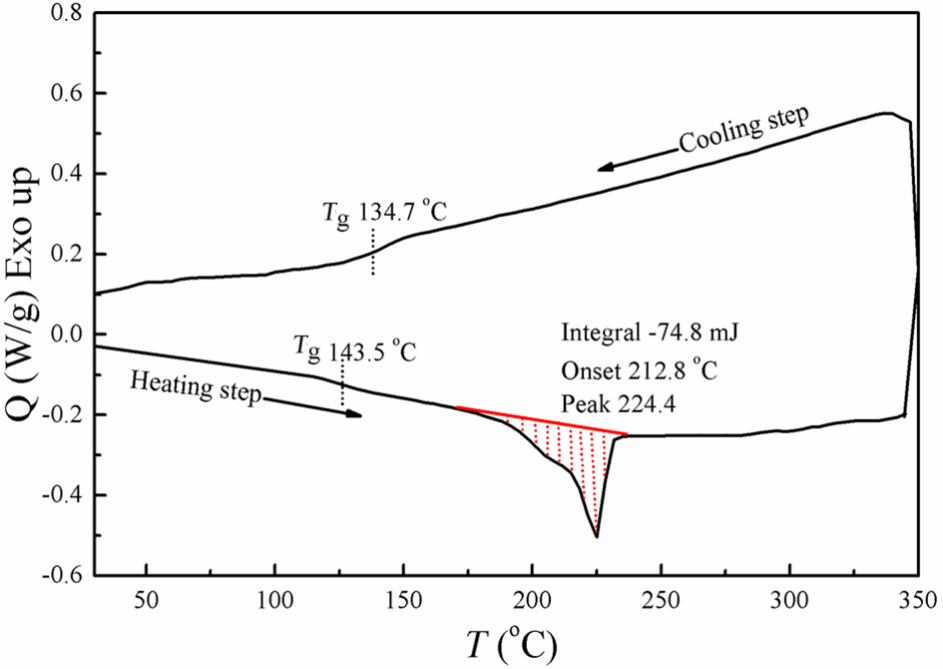
DSC profile of the PC/MWCNT film at 1 wt % loading.

Figure 6 shows the DSC analysis of the PC/MWCNT film with 1 wt % loading, in both heating as well as cooling cycles. The DSC curve of the nanocomposite in the heating cycle exhibits a glass transition temperature at ≈143.5 °C. No amorphous polymer can exhibit a melting transition, as melting is a first-order transition occurring only for crystalline polymers. However, an endothermic peak followed by a shoulder with the enthalpy heat of fusion (27 J/g) is observed at 224.4 °C. This might be due to the possible ordered structure or segmental chain mobility of PC macromolecules changed due to the interaction of the MWCNTs. The lack of a cold crystallization peak appearing in the cooling cycle could be due to the enthalpy relaxation or mesophase transitions. The glass transition observed in the cooling cycle is 9 °C less than the heating cycle.

## Conclusion

PC/MWCNTs composites were prepared as transparent films. The presence of a dispersed, uniform phase in the composite films was confirmed. Vibrational spectroscopy was used to assess the change in behavior of the polymer matrix when adding the selected fillers and to discriminate the crystalline/amorphous balance behavior induced thereof. The fillers decreased the crystallinity as compared to the bare polymer. Thermal analysis allowed interpretation of the effect of filler loading into the PC matrix in terms of thermal stability, which was significantly enhanced with respect to the bare polymer. It can thus be speculated that MWCNT fillers influence the likely solvent-induced crystallization in PC. We think that the demonstrated study is promising and could help the research community in this area. The investigated composites, upon further characterization (e.g., by thermocycling or other artificially accelerated aging protocols), could possibly be a candidate for replacement of bare PC in a variety of applications.
